# Breaking the silence: A qualitative exploration of parental perspectives of children with Goldenhar Syndrome

**DOI:** 10.1016/j.heliyon.2024.e24328

**Published:** 2024-01-21

**Authors:** Rebecca Hitchen, Maxine Woolhouse, Patricia Holch

**Affiliations:** Leeds Beckett University, Psychology, School of Humanities and Social Sciences, Portland Building, City Campus, Leeds, LS1 3HE, West Yorkshire, UK

**Keywords:** Goldenhar syndrome, QoL (quality of life), Rare condition, Genetic mutation, Stigma, Uncertainty, Group support

## Abstract

**Background:**

Goldenhar Syndrome is a rare congenital condition, typically characterized by craniofacial abnormalities and vertebral malformations. Due to its rare and complex nature, the etiology is unconfirmed, resulting in parental uncertainty and subsequent emotional sequelae. Clinical manifestations have been researched but few studies have explored parental wellbeing and Quality of Life (QoL). In this qualitative study, we explore parental views of the challenges and lived experience of raising a child with Goldenhar Syndrome.

**Methods:**

Ten biological parents (five mothers and five fathers), recruited at the Goldenhar UK Conference, took part in audio-recorded, semi-structured interviews. Interviews explored emotional wellbeing, views surrounding causation, support accessed, challenges faced, experience of stigma and future outlooks. Reflexive thematic analysis was employed, and transcripts were subject to deductive and inductive coding.

**Results:**

Seven themes were identified: support networks (Goldenhar UK), rollercoaster of emotion; gendered coping; uncertainty; societal reactions; coping with challenge and acceptance.

**Conclusions:**

This is the first-time the life perspectives of parents, raising a child with Goldenhar Syndrome, have been explored via interviews. We have unearthed prominent issues that impact parental QoL including isolation and distress at the point of diagnosis, and throughout the multidisciplinary health journey. We have also established significant indicators of the ongoing QoL challenges faced by young people with Goldenhar Syndrome. Future work is underway exploring these issues further with teenagers, young people and adults with Goldenhar to develop a conceptual framework of their QoL. This will be used to develop a bespoke patient reported outcome (PRO) to give voice to the challenges children and young adults face during their medical journey.

## 1 Background

Goldenhar Syndrome is a rare condition, first identified in 1952 by Dr. Maurice Goldenhar, a Belgian ophthalmologist [1]. Goldenhar Syndrome (GS) can cause a wide range of soft tissue and bone abnormalities affecting the face, the vertebrae and other body parts. Seventy to eighty percent of cases affect one side of the body or face [2].

A child with Goldenhar Syndrome may have a range of unique features including; the under-development of one ear, appearance of skin tags, malformations of the middle-ear canals and hearing loss, eye abnormalities, cleft lip and/or palate, wide mouth, underdeveloped jaw or cheek bones and misplaced or missing vertebra. Goldenhar shares characteristics with; Hemi-facial Microsomia (HFM), Craniofacial Microtia (CFM) and Oculo-Auriculo-Vertebral-Spectrum (OAVs); and it is often used as an ‘umbrella descriptor’ for these conditions [3]. Goldenhar also has parallels with DiGeorge Syndrome/Velo-Cardio-Facial Syndrome/22q11.2 Deletion Syndrome. When facial differences are the only noticeable difference it is normally referred to as Hemi-Facial Microsomia (HFM), but when associated with abnormalities of the vertebrae, heart and kidneys it is referred to as Goldenhar Syndrome (GS) or OAVs [4,5]. Goldenhar should not be used interchangeably with HFM [6]. It is important to note, children with Goldenhar may look slightly different, but these physical differences do not affect their intelligence or their ability to live happy and fulfilled lives.

### 1.1 Prevalence

Overlapping diagnoses make it impossible to get a true picture of Goldenhar incidence [7]. Considerable discrepancy exists in reported prevalence rates of Goldenhar and OAVs, ranging from 1 per 5642 to 1 per 45,000. EUROCAT, (Europe's population based congenital anomaly registry) [8] claims there are 3.8 per 100,000 OAVs births per year [9] and Shrestha & Adhikari (2015) [2] report a prevalence of 1 per 25000–45000 births. Research continues to report discrepancy over OAVs rates; ranging from 1 per 3500 to 1 per 45,000 live births. Male infants are more commonly affected with OAVs than females with a 3:2 ratio [10]. Variation of rates differ so vastly because the spectrums of these overlapping conditions are so broad e.g. HFM is relatively common, but OAVS and GH are not.

### 1.2 Causation

Due to the complex nature of Goldenhar, the underlying etiology is unknown, and as cases appear to occur sporadically it is still considered a world-wide enigma. There have been innumerable diverse, contradictory, and inconclusive suggestions for causation [2]. These include environmental factors such as pesticides, influenza, viruses in pregnancy [3] and auto-immune deficiencies [11]. The NHS site, Patient Info makes links to drug use in pregnancy (cocaine, thalidomide, tamoxifen) and it also implicates maternal diabetes and rubella [12]. Overdoses of retinoic acid (Vitamin A) suggestions have been documented [13]. Gulf War connections have also been suggested; however studies have demonstrated no significant increase in the odds of GS amongst military serving parents [14].

Some studies have reported familial transmission [15] suggesting this leads to autosomal recessive inheritance (two copies of an abnormal gene being present) or dominant inheritance (an overly dominant gene causing a mutation). Multifactorial inheritance (genetic and environmental factors) has also been proposed [16]. Terhaar's 1972 [17], discovery of monozygotic twins concordant for OAVs pointed towards a genetic explanation for this syndrome.

A growing body of literature is proposing a genetic predisposition [18]. Many studies have identified a gene located in the 22q11 chromosome region to be potentially responsible for signaling events, modulating a spectrum of change, partial gene deletion or doubling [19,20]. Mcdonald-McGinn et al., 2023 [21], explain that Velo-Cardio-Facial Syndrome and DiGeorge Syndrome were originally thought to be separate disorders before a deletion in chromosome 22q11.2 was identified in individuals affected. Goldenhar is currently unconfirmed but thought to share similarities [21]. The UK charity, Unique, international provider of information on rare chromosome disorders, also describe chromosome 22q11.2 deletion as Velo-Cardio-Facial-Syndrome [22]. Unique also provide information on 22q12 and 13 duplications and Cat Eye Syndrome (CES), both of which have Goldenhar parallels [23,24]. Unique specifically state ‘whether duplication or deletion is inherited or de novo, no dietary, workplace or lifestyle factors are known to cause these chromosome changes’ [22].

Until January 2022, Harmony DNA pregnancy screening tested for abnormalities/micro-deletions in 22q11.2 (DiGeorge Syndrome) at 10–14 weeks but discontinued this test due to reliability issues and uncertainty surrounding small, nested deletions [25]. Around the world there is huge variation in testing, for example, Hong Kong's extensive DNA test, Prenatal Peace, screens for trisomies (an extra chromosome) in 21,18,13,22,9,16 and offers a further test for diagnoses of structural chromosomal alterations and micro-deletions conditions such as DiGeorge Syndrome (22q11.2 deletion); this test is also used in New Zealand. The Swiss genetic testing company, Swissgenoma, also screens for conditions with structural alterations and microdeletions such as, 22q11.2, 11q23 etc in their PrenatalSAFE test. As 22q11.2 Deletion has parallels with Goldenhar Syndrome, perhaps these tests may provide useful insights in the future.

Singhal & Tripathy (2023) [10] also propose abnormalities in 22q11 and a partial trisomy of the 22q11 region; other chromosomal abnormalities they suggested are mutations of region 15q26 and mosaic trisomy of 7 and 9. Benbouchta et al. (2021) [26] claim 15q26 deletion could be related to growth restriction, heart defects and skeletal abnormalities. Mosaic trisomy 8 describes some symptoms which share similarities to Goldenhar [27]. Also, functional studies suggest that an over dominance of the gene MYT1 (chromosome 20), associated with developing the nervous system, may down-regulate and disrupt all retinoic acid receptor genes and signaling pathways [28]. Retinoic acid receptors regulate genes associated with normal vertebral cellular processes, cell proliferation and embryonic development. Timberlake, A et al. (2021) [29] report Haploinsufficiency of gene SF3B2 (chromosome 11q13.1) to be the cause of HFM and Goldenhar. Haploinsufficiency occurs when one copy of a gene is inactivated or deleted [29].

Despite increasing evidence of genetic claims, universal agreement has still not been reached due to the complex and heterogeneous nature of the syndrome, and a recent review recommends more studies are needed to explore how genes mutate and interact with environmental factors [30]. Microdeletions of genes are relatively common and more research into Goldenhar microdeletions and duplications is needed, as a growing body of evidence appears to suggest that Goldenhar could result from mutations in 22q11 (microdeletions in 22q11.2, duplications in 22q12 and 13 or possibly (CES) [[22], [23], [24]], in combination with mutations from other proposed chromosomal areas, creating a unique chromosomal umbrella. This individual, genetically diverse, chromosomal umbrella could provide an explanation for the huge variations within this syndrome and the uniqueness it creates.

### 1.3 Treatments

The varied and complex etiology means treatment for Goldenhar Syndrome is multi-dimensional and clinicians recommend an individual approach supported by a multi-disciplinary team including a range of medical professionals such a neonatologists and pediatricians [31]. Parents and children face multiple appointments throughout the stages of their Goldenhar journey (dependent on the severity of the syndrome) examples include; ophthalmological removal of dermoid cysts (on the cornea/sclera and coloboma), otolaryngological consultations for hearing loss and orthopedic appointments for scoliosis realignment with vertebral braces [32]. Children and young adults sometimes face multiple surgeries (including orthodontic treatment as a precursor to maxillofacial surgical alignment procedures) [33]. There are not only physical challenges for children but also there are clear, serious psychological and social challenges of living with facial difference; there is a paucity of work in this area [34,35]. These psychological challenges are often exacerbated by public misconceptions.

### 1.4 Public misconceptions and stigma

Facial disfigurement has been recently described as a globally neglected human rights issue [36]. Over 1.3 million people in the UK (1 in every 124 school children) live with facial disfigurements (scars, birth marks, skin conditions or congenital syndromes) [37]. Despite this prevalence, the ‘Disfigurement in the UK’ report discovered 49.5 % of children with a facial difference experienced appearance-targeted bullying at school [38], findings are also reported in previous research [39]. Discrimination is expressed through attitudes and behaviour, including social challenges and lower expectations at school and the workplace [40]. These social challenges and stigma can have a debilitating impact [[41], [42], [43]]. Changing Faces recognize these barriers and fought to get face equality into the National Curriculum [44], and they created classroom resources such as Wonder and A World of Difference [45]. Changing Faces advocate positive media progression and believe the media have a responsibility in their portrayal of facial difference [46].

### 1.5 Media representation

The media have historically portrayed people with facial disfigurements in a negative light, for example Freddie Kreuger in ‘Nightmare on Elm Street’ and consistently the ‘James Bond’ villains, [47]. Indeed, there is a general negative and sensationalised attitude towards disfigurement in the media [48]. However, things are slowly changing. In 2018, the British Film Institute stopped funding movies in which villains appear with facial disfigurements following a campaign from Changing Faces called ‘I Am Not Your Villain’ [49].

R.J Palacio's famous book, *Wonder* (Palacio, 2012) [50], provided a positive example of representation in the media. The 2017 *Wonder* film uses the headline catch-phrase ‘*You can't blend in when you were born to stand out’*. The story features a ten-year-old boy displaying a HFM characteristic of OAVs Syndrome experiencing discrimination at school [50],which brought Goldenhar Syndrome to the spotlight. *Wonder* educational resources are currently being used in UK schools. Other recent effective interventions include personal narratives by individuals with facial disfigurement (showing themselves as socially confident and emotionally stable) enhancing perceptions of their skills and personality [51]. A positive Goldenhar life story was recently broadcasted on UK media (BBC Look North) [52], and also GH UK members can view many positive stories on Goldenhar UK Facebook and Instagram accounts.

### 1.6 Stress on children and parents

It is clear the diagnosis and treatment of Goldenhar can put considerable stress on children, their parents, and the whole family. Rare disease studies highlight certainty as a primary parental care need [53,54]. The unexplained causation of the syndrome produces profound uncertainty for parents resulting in rumination and a pre-occupation with what happened and why [55,56]. This can contribute to emotional changes such as anxiety and depression [57]. Although no work to date has explored the impact of Goldenhar Syndrome on children and parents, one study surveyed HFM patients and their carers, who identified negative social experiences, multiple medical stressors and suggested health care providers need to be better informed about Cranio Facial Malformation (CFM), suggesting there should be greater coordination among specialists, and that a family-centered approach should be used [58]. Interestingly, a study exploring the QoL of parents and children with HFM (compared to matched controls) found that there was no difference between the matched control and HFM children's scores however parental assessment of their HFM child's QoL was significantly worse particularly for physical, social, and school functioning [59]. This suggests that parents feel their children's needs acutely and need information and support to buffer this.

### 1.7 Parental support

Findings suggest parents of children with rare syndromes often feel silenced [60] and lack medical advice and social support [61]. Research indicates this can lead to isolation and have psychological implications including anxiety [62]. Support groups help foster meaningful relationships and provide psychological benefits [63], this is particularly important for parents of children with rare conditions [64,65]. This support, actual or perceived, consistently improves mental and physical health, increases self-esteem and buffers stress [66]. Bogart & Hemmesch (2015) [67], found an association between repeated conference attendance for parents and children suffering rare syndromes and increased self-efficacy, concluding rare syndrome support conferences are promising QoL interventions.

### 1.8 Aims

Goldenhar is a rare condition and parents feel isolated with the uncertainty around the causation and the challenges their children face living with physical and facial difference. These challenges are compounded by the way facial differences are inadequately portrayed in the media, and also compounded by a lack of understanding from the public, placing parents under significant stress. Thus far, in the published literature the impact of Goldenhar Syndrome on parents is yet to be examined. Here we aim to explore via qualitative interviews to gain a richer understanding of:parental responses to initial diagnosis and causation, experiences of stigma, discrimination and challenges faced, parental perceptions of societal awareness, changes in parental perspectives over time and lastly their perceptions of support received within the Goldenhar UK network.

## 2 Methodology

### 2.1 Participants and recruitment

Ten participants were recruited, an equal balance of five mothers and five fathers to explore potential gender differences in experience. Participants were recruited as a volunteer sample via an invitation letter from the Goldenhar UK group. Inclusion criteria required participants to be over eighteen and a biological parent of a Goldenhar child.

### 2.2 Design

This was a cross-sectional qualitative semi-structured interview study. A qualitative approach was adopted to enable a full exploration of experiences, attitudes, and behaviours, which can be difficult to capture quantitatively [68]. We employed a direct-realist approach to enable focus on lived-experiences, thus using ‘*people's words to provide direct access to reality’* [69], p224). A post-positivist approach was selected as the research paradigm to account for possibility of researcher bias [70].

### Ethical approval

Ethical approval was obtained from Leeds Beckett University Psychology Ethics Committee on May 12, 2017 (number RH/PH/AW/120517) and the study was conducted in accordance with the British Psychological Society's code of ethics [71].

### 2.3 Materials

A semi-structured interview schedule was developed to focus on topics highlighted from previous literature of parents with children with facial differences and rare conditions. These included experience of the initial diagnosis, parental emotional wellbeing, causation confusion, challenges they faced, experience of stigma, coping strategies, support at home, how Goldenhar UK and the conference weekends help them, and the adjustment of feelings and perceptions over time.

### 2.4 Procedure

Interviews were conducted by the first author (RH), at the Goldenhar UK Conference in May 2017. Informed consent was obtained, parents were reassured of their right to withdraw and right to avoid answering any questions with which they were not comfortable. Interviews lasted approximately 30-min and took place (in-person, in English) in a private room with only the interviewer and parent present, interviewees were fully debriefed afterwards.

A semi-structured approach was adopted to allow for flexibility and provided opportunity for impromptu revelations. The recordings were stored securely on a university recommended OneDrive and deleted after transcription. Interviews were transcribed verbatim, accurately reflecting what participants said irrespective of accent, local dialect, grammatical and syntactical errors. Pseudonyms were used in transcription to ensure participant anonymity and confidentiality.

### 2.5 Data analysis

Braun & Clarke's reflexive thematic analysis (TA) was employed [[72], [73], [74], [75]], providing an accessible, systematic, and rigorous approach to coding and theme development, questioning conceptualisations, and experiences, summarizing and classifying participant's accounts and making sense of participant's contributions [76]. See Table 1 for the 6 stages of thematic analysis; familiarization, generating initial codes, searching for themes among codes, reviewing themes, defining, and naming themes, and writing the final report. A hybrid deductive and inductive approach was adopted, employing both latent and semantic coding. In practice, this involved listening to the recorded interviews several times to form accurate interview transcriptions. It involved rigorous reading, familiarization with the data, and systematic coding which involved highlighting key segments and phrases that were relevant to the research area. Comparative coding across transcripts took place to explore the commonality of themes. Themes that clearly identified a pattern of meaning were clustered together, colour-coded and used as the initial building blocks of our initial thematic map. Themes were reviewed and refined numerous times to ensure the label fitted the description of the story. Finally, the writing up phase involved weaving together the analytic narrative and data extraction and then contextualising the analysis in relation to relevant literature. A colour-coding system was adopted to group themes and initial sub-themes resulting in an initial thematic table (see supplementary material 1), finally overall themes were reviewed and refined.

**Table 1:** The six stages of thematic analysis (Braun and Clarke, 2006).

Stage	Description of analysis process
**1. Familiarization**	Transcription of data, re-reading data and noting down initial themes
**2.Generating initial codes**	interesting features of the data in a systematic way across the whole data set, collating data relevant to each code
**3. Searching for themes**	Collating codes into putative themes, gathering all relevant data to potential themes
**4. Reviewing themes**	Checking if themes work in relation to coded extracts and the full data set, reviewing data to search for additional themes, generating a thematic map.
**5. Defining and naming themes**	Refining each theme, generating clear definitions and names for each theme.
**6. Producing the report**	Selection of illustrative compelling extracts, final analysis of selected extracts, relating analysis back to research aims and literature

**Fig. 1:**
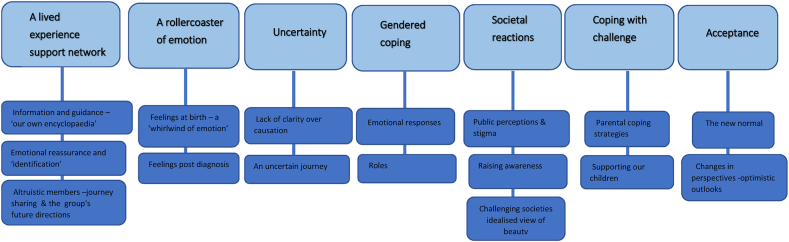
Themes and sub themes (thematic analysis).

Please see Fig. 1 for a summary of the themes and sub themes identified in the thematic analysis.

## 3 Results

### 3.1 Participants

Ten parents of children with Goldenhar Syndrome (5 fathers and 5 mothers) age range 34–51 (mean 41.5: SD 5.50), their children's ages ranged from 3 to 19 yrs (Mean 10.6: SD 5.73). All participants were married.

### 3.2 Goldenhar UK – 'a lived-experience support network’

#### 3.2.1 Our own encyclopedia

All parents were positive about the information, advice and support they received from the Goldenhar UK Group. Sonia described it as *“our own little google*” and “*more beneficial than an internet webpage because it is what people have dealt with.”* Bruce explained that group advice helped his son with ear construction decision-making *“not knowing about the group we would have gone down another avenue.”* Jasmine explained how she had turned to Goldenhar UK for advice over the years and described her experience as, “*Priceless! Absolutely priceless ….you get to see exactly what the future holds.”* She also explained it was also important to share knowledge explaining “*you've got new families coming … you can impart some knowledge to them*.” Jules found the group helpful and insightful for her journey with his Goldenhar child.

“They have been a massive help just with information …, it gives me a very good look from where my boy is at -to where he might be going in the future, for like his health and stuff. (Jules)

#### 3.2.2 Reassurance and identification

Identification with others and peer-reassurance were identified as key benefits of conference attendance. Maximus and Jasmine describe how parents are at different journey stages, “*we are all helping each other out.” “We have all been going through a similar sort of journey … we all know the ups and downs … … peaks and troughs of operations …..appointments … it is nice to know … you've got other families in the same boat, and you can talk …. it means a lot. (Jasmine)*

Being in the “same boat” enables families to discuss parallel stories and supply peer-support. Sonia described how Goldenhar UK was *“reassuring for the new parents and for any parent that hasn't discussed the possibility of having that operation with their child.”* Jules reported that talking to the group chairperson ‘*eased his mind.’*

Chloe considered identification with others to be the most important aspect of Goldenhar UK conference attendance:

*“ We like to see my daughter feeling like she has other friends who are going through something similar … … … she started to notice her own facial differences … she would ask why she had a little ear and why her face was crooked …. we recognised … it would be nice for her to know … she is not the only child with those facial differences.”* (Chloe)

Many parents also commented on the importance of identification with others stating that it provided validation for their child and lessened feelings of anxiety and isolation.

#### 3.2.3 Altruistic notions and future directions

Altruistic desires to support and advise others were expressed by parents. For example,

Maximus declared his eagerness to explain school statementing processes of educational need to help parents access full support. Michael and Jules expressed desires to be involved in the development of Goldenhar information leaflets. They both volunteered photographs and Michael said, *“we want to write … about our daughter and … the journey … for that booklet”* (information leaflets)*.* Michael suggested *‘peer-mentoring’* for children as a future direction and other proposals from Lois, Judy and Michael were inviting older Goldenhar people to future conferences.

### 3.3 A rollercoaster of emotion

#### 3.3.1 The birth –an emotional whirlwind

The interviews revealed all parents went through healthy pregnancies without cause for concern. Luke and his wife went through “*all of the pre-natal cares … scans .. but there was no hint.*” Nine out of ten parents found their first encounter with their newborn child to be a shock. Bruce, described his first meeting as surreal:

*“It was a shock! I was scared it wasn’t a happy occasion at the time because we were told he wasn’t going to make 24 hours, so it was like kind of a blur kind of a dream not believing it was happening”.* (Bruce)

Parents expressed emotions including ‘*shock,’ ‘disbelief,’ ‘anger,’ ‘confusion,’ ‘stress,’ ‘grief’* and feelings of failure. Judy describes her emotional anguish:

*“I went through … grief ... … everybody expects to have a child without any additional needs. I went through …... disbelief …. anger as well, why did it happen to us? Why did it happen to my son!? What have I ever done wrong?”* (Judy)

Sonia also likened the experience to grief because her baby “*was in hospital rather than being at home where he should be*.*”* Jasmine described the experience as “*a complete whirlwind”*:

“It went .. to finding a catalogue of things ….. a big list by the end of the day we had about ten things. It went from I can cope with that, I can cope with that, I can cope with that, oh my gosh how much more!?” (Jasmine)

Michael, uniquely, was not shocked and stated that he was not concerned about aesthetic difference but was worried about others' reactions, *“the nurse very tentatively pointed out her little ear … we looked at each other and said .. we don't care. I suppose it is other people's reactions more … you feel … you have to explain*.”

#### 3.3.2 Feelings post-diagnosis

The timescales of diagnosis ranged from birth to eighteen-months and sometimes parents reported diagnosis not being that helpful because as Maximus explained, *“you don't know what Goldenhar is, it not something everyone has heard about.”* Jules's extract illustrates this further:

“As soon as he was born, they told us the name of the diagnosis and they didn't give us much information I don't think they knew where to start! They just had to go and look online. I decided to Google this thing”. (Jules)

The lack of perceived knowledge by medical professionals added to parents' confusion. This led to frustration; parents were ‘googling’ the rare syndrome and doctors appeared to be researching on the job. Bruce reported that doctors “*start to learn from each other … how to do these certain procedures*.” He stated that it was *‘an emotional rollercoaster’*. Many parents reported feeling *‘overwhelmed’* by the volume and complexity of the medical information they were bombarded with. Jasmine exclaimed, “*they … assumed I knew quite a lot and I didn't know anything*.”

### 3.4 Gendered differences in coping

#### 3.4.1 Emotional response

Findings suggested that some gender-specific differences in emotional responses were apparent. Self-blaming in mothers was evident, four-out-of-five mothers blamed themselves initially. Phrases such as, ‘*I must have done something wrong’, ‘everybody is going to think … I've done something’ or ‘it's my fault’, ‘all of a sudden you discover that you haven't done everything right’,* were expressed by mothers.

Lois's extract clearly depicts her feelings of guilt and confusion.

“Why me!? The first question, what have I done wrong? And I started thinking back, did I take pain killers? I didn't drink alcohol. Did I fall? … there was loads of questions, loads of questions blaming myself at the time.” (Lois)

Mothers spoke openly, revealing feelings of ‘*self-blame,’* ‘*grief’* and *‘emotional disbelief.’* Judy explained how her first reactions were catastrophic and the experience sent her a bit ‘*crazy’;* “All of these things, they go through your mind … they can … send you a bit … crazy.” Chloe depicted her initial feelings of failure as bizarre, “I *was elated … I had a baby! So, after my initial thought of oh my mum is going to kill me, I was absolutely elated.”*

Jasmine described how her eldest (non- Goldenhar) son helped her avoid isolation:

“I think if I didn’t have him then I probably would have isolated myself for a long time. I wouldn’t have pushed myself to go out and about ..because it was dealing with other people’s questions and other people’s looks.” (Jasmine)

Sonia and Lois also expressed initial desires to isolate themselves to avoid judgement and questioning. Many mothers felt angered by others' reactions and found negative experiences contributed to feelings of anxiety and self-blame. Jasmine explained that *‘looks’* were sometimes hard to deal with, and the assumption “every time you … went out .you had to talk about it” was ‘emotionally draining.’ Chloe, however, found she could explain it to her husband and people *‘without any issue.’* Chloe reported the first five days being “*more harrowing*” for her husband because she was never separated from her baby. She exclaimed that “*he cried every night, but I didn't really cry at all, I had no major upset*.” Judy also referred to her husband's emotional battle during surgery describing him as “*very strong*” on one side but “*stuff like that he can't deal with*.”

In contrast to the mothers, none of the fathers expressed feelings of self-blame or failure. Most fathers appeared to have adopted a ‘hands on’ pragmatic approach:

“*For me it was different to my wife … he has Goldenhar we have got to get on … it is a bit more cold and hard facts for me … It was put the emotion to one side and let’s understand what we need to do to move forward*.” (Maximus)

Maximus's extract illustrates his *‘put the emotion to one side’* clinical approach. Later in interview Maximus revealed that *‘in the back of his mind he sometimes thought like his wife,* but he reiterated “*we can't change it*” and you have to “*deal with it … just treat every day as it is*.”

Luke also adopted a pragmatic approach and was reluctant to acknowledge his emotions. When asked how the experience impacted upon him emotionally, Luke responded by saying “*I've always been a fighter .. …. my parents were …. very good parents … I think they gave us very strong …, armour to deal with the real world*”. With his ‘*masculine armour,’* Luke shifted the focus from himself and deflected it to his wife:

“I didn't think of it as a big issue …... I was just so glad that she was okay, and my wife was okay …. my wife, is very smart … although, .. she did need other reassurances from the medical field and slightly more support.” (Luke)

Michael also focused on his wife “*she wanted to make sure that she had done the things that she could have during the pregnancy.”* He illustrated how he supported her by saying “*she was as healthy as she could be, and she was absolutely spot on.”*

Contrastively, Jules and, Bruce both talked openly about their emotional experiences:

“They didn’t know what was wrong … telling us a list of things about what’s wrong with him … saying …. we don’t think he is going to make 24 hours … it was a … very very scary experience, very emotional as well.” (Bruce)

Like Bruce, Jules also revealed how ‘*scared’* he felt and how he feared his son would be bullied. He described lengthy periods at the hospital as *‘emotionally tough.’* Bruce described how he felt robbed of his experience of being a new father and consequently his intense fear of future problems led to his absence at his daughter's birth, “*it affected me a lot … I was scared to go to that one just in case.”*

### 3.5 Gendered roles

All parents said they were supported by their spouse and worked together. Judy said her husband's pragmatic approach helped her to cope, “*My husband and I complimented each other really well because I was probably an emotional wreck … at the very beginning*.”

Overall mothers dealt with more of the appointments, Jasmine exclaimed *“the appointments side is my sort of demeanour.”* Three fathers described how their wives were responsible for online Facebook communication. Mothers also appeared to have a higher responsibility for dealing with other people's questions and reactions. Extra medical and emotional considerations combined with juggling motherhood and working full-time caused an *‘inner-battle’* over work-life balance for mothers, Judy and Chloe. Chloe reported how *“it is harder for families … with issues. ….other people judge you a little bit more harshly.”*

### 3.6 Uncertainty

#### 3.6.1 The causation conundrum

The causation question baffled all parents at certain points and continued to concern some. Bruce described how *“it is really difficult for them to pinpoint what causes it*.” This unclear situation provoked mixed responses from parents who expressed more concern in the initial stages, particularly mothers. Judy said that “*the why question lasts a really long time”* and she just wanted to know why. Other parents accepted that they will never know why. Michael, was fairly happy and stated, “*we will never really reach a definitive understanding of what it is, and it doesn't really matter.”* Jasmine was also happy that a cause had not been identified and said it was not important anymore. Maximus said lack of clarity was not an issue and described himself as being *‘fine with it’* stating, “*there are a lot of unanswered questions out there … and this is just one of them!”*

Sonia communicated the fact that an explanation wouldn't change anything, but clarity would aid understanding and provide future reassurance for children and their families:

“For my child’s point of view … if they go on to have children of their own … you would like to give them that little bit of reassurance … unfortunately you were born due to this, this, or this but you can’t give … reassurance because you don’t know.” (Sonia)

Lois's response mirrored Sonia's. Additionally, Bruce highlighted how a lack of explanation makes it difficult for parents who want more children because *“you don't know if you are doing anything right.”*

#### 3.6.2 An uncertain journey

Parents described Goldenhar as an ‘*uncertain journey’* because there is so much confusion over the condition and uncertainty over its progression. Lois reported that after numerous tests medics were unsure and couldn't tell her whether *‘it can get worst or better.’* Parents expressed strong feelings of uncertainty about surgical decision-making, uncertainty surrounding their child's psychological wellbeing and uncertainty about how to support their child. Chloe's extract clearly outlines why making an informed decision is such a challenging task for parents.

“You feel overwhelmed … no amount of biological background can help you make those decisions … the condition itself is rare … we are dealing with such a … small cohort … outcomes from … surgeries … can be great for one person and not great for another …, there are not the numbers to say what is statistically the best option .. I struggle like any other parent when it comes to making decisions about what is … the best surgical outcome coupled with what is going to be the best emotionally for your child.” (Chloe)

Without medical evidence or a comparative sample, families must decide and hope for the best. Michael pointed out how difficult the weight of surgical decision-making can be for children. He also described how respecting your child's decisions and being *‘as neutral and unbiased as possible’* is tough.

Parents worried about how their child would cope in surgery and some parents worried about their transmitting their anxieties onto their child. There was also uncertainty about their child's future happiness:

“I don't know how his family life will affect him ….we do get a bit worried ….Will he be able to have children? Will he be scared to have children? Because I don't think there are any studies developed in this way …. if they are born with Goldenhar.” (Lois)

### 3.7 Societal reactions to goldenhar

#### 3.7.1 Public ignorance and stigma

Interview responses showed that many parents had experienced stigma in terms of feeling ‘othered’ such as stares, uncomfortable silences, intrusive questioning, hurtful comments, and unhelpful assumptions. Sonia described how others *‘assumed the worst’* and interrogated her*:*

“I was constantly being stopped … I …. purposely made little cards it said … I was born with Goldenhar Syndrome if you want … more information here is the details … the next person who asked me what was the matter with my child I just gave them a card and said, they will tell you all of the information you need –goodbye”! (Sonia)

Sonia clearly illustrates how stigma and public ignorance affected her and how information cards were a useful strategy. Jasmine explained how she wanted to socialize normally but found it difficult because *‘people have too many questions.’* Bruce experienced ‘*talking down’* and unfair assumptions about *‘what is best’* being made at school. Lois communicated the fact that nobody said anything to her, but she still felt stigmatised.

#### 3.7.2 Raising awareness

Interview responses suggested parents felt that in society physical differences were starting to become more widely embraced, with Michael describing diversity shown in the UK CBBC My Life programmes (a documentary series following the highs and lows of unique children across the world), (Childrens BBC, 2023) [77]. However, parents still indicated that there is a clear lack of public awareness surrounding Goldenhar and they would like to see more representation and normalisation in the media.

Bruce exclaimed, “*if it was more in the limelight .. it would .. help us as parents and help the children”.*

Many parents expressed how excited they were about the release of the film, *Wonder*. Positive comments were voiced such as ‘*I was elated*’ and ‘*it's really good*’. Jasmine exclaimed, *“It's very much like my child … the operations ... … when I looked at it, I thought, gosh that could be him …. When I saw it advertised, I thought wow … everyone … will see the challenges … these little people have to go through, ….and the parents …. it is nice … there is going to be a film promoting that.”*

Many parents hoped the film would raise awareness and tackle societal disfigurement discrimination. Parents also worried about *‘issues it might raise’* for their child. Michael challenged the fact that the film was not using an actor with a real facial difference, stating he *“would have loved to have seen somebody with a disability actually play that part.”*

#### 3.7.3 Society's idealized beauty views

Parents expressed concerns about the discrimination caused by society's obsession with *‘perfection’* and its narrow projection of ‘so-called’ beauty images:

*“You are bombarded by society of images of what is perfect … what people should look like … it made me feel sad that my daughter has to … make her way within a society where you have got that sort of consistent prejudice.”* (Michael)

Chloe felt particularly irritated by the media's obsession and constant presentation of ‘*the perfect baby.’* She felt ‘perfection messages’ were not helpful messages to be delivered to our children.

### 3.8 Acceptance

#### 3.8.1 The new normal

With time, parents’ acceptance of Goldenhar occurred and it became normalized.

Maximus said, *“Goldenhar is normal. It's just something slightly different that's all!”* Judy often forgets her son has Goldenhar, “*it's only really when I come to these events (Goldenhar family conference) that I realise he has Goldenhar.”* Jasmine explained how “*it used to be tears … every time someone would talk about it but now … it's just part of our life and … we're trying to be like any other family now.”*

#### 3.8.2 Positive outlooks

Many parents explained that despite the challenges they faced, Goldenhar had ‘*enriched’* their lives and changed them for the better. Parents acknowledged the shift in their outlook and viewed the future in a positive light:

*“Clichéd but it has made me take a long hard look at myself and what I valued before having children … we are happy, my expectations have changed ….all I want out of life now is just to be happy, …that doesn’t mean having a million holidays … driving fancy car ….just ….have time for each other. We are certainly not miserable at all, and we always try and find the positive in everything.” (Chloe*)

Chloe outlines how her life expectations have changed and how family time is more important than material goods. Judy described how having her son “*was a great joy*” and how she “*met some wonderful people and most probably … changed as a person.”* Bruce explained how his family became stronger and now depicts himself as “*more of a proud dad than a scared dad*.”

## 4 Discussion

This study is the first to explore the experiences of parents who have a child with Goldenhar Syndrome via semi-structured interviews. Analysis identified seven key themes: a lived-experience support network, the rollercoaster of emotion, gendered differences in coping, uncertainty, societal reactions, coping with challenge and acceptance.

The findings illustrated that in general parents experienced initial shock at the diagnosis and expressed a range of feelings, indicative of cognitive disruption and rumination. This mirrors the predicted response to uncertainty identified by Pennebaker et al. [55], and demonstrates the importance of a sense of certainty as an essential primary care need for parents [53].

Most mothers interviewed experienced self-blame and research shows parents caring for children with rare conditions often report this [61]. The self-questioning and mother-blaming is supported by feminist theory [78], encompassing expectations and idealisations of the mother as the primary care giver of a child [79]. This often starts before birth, where a mother's individual influence over a vulnerable fetus is frequently emphasized and all aspects of her life are scrutinized [80]. For example, working mothers of anorexic daughters are blamed and branded selfish and accused of rejecting the traditional values of motherhood [81], and mothers of autistic children have been labelled ‘refrigerator mothers’ [80]. There is no surprise that this mother-blaming phenomenon and obsession with ‘good mothering’ contributes to the guilt and blame mothers feel.

Also, it is not surprising that most fathers did not appear to share feelings of blame or guilt and opted for pragmatism, suppressing their emotion and projecting this onto their wives [82]. The reluctance to discuss emotion and shift focus to their wives mirrors masculine ideals which reinforce self-control, rationality and reject emotional vulnerability [83]. Contrary to this, two fathers did discuss feelings of fear, breaking down this stereotype [84], and challenging hegemonic masculine ideals [85].

Although parents reported working cooperatively and supportively, it was evident that mothers appeared to have a higher mental-load, for example, more responsibility for information sharing, appointment attending and dealing with other people's reactions and questions. Gender schema theories in heterosexual parenting largely reinforce parenthood as a woman's domain [86]. Thus, women shoulder the extra burden of childcare responsibilities and family health work. This extra burden contributes to career sacrifices for mothers; reduced hours, taking on a ‘second shift’ after work or potentially penalizing their careers [87]. Gender schemas suggest men's stress frequently arises from work demands while women often report greater stress from personal and family responsibilities. The conflict over responsibility for work and family roles can leave mothers with a sense of guilt [86].

The Goldenhar diagnosis appeared to confuse both parents and medical professionals. Initially this caused uncertainty, stress, and frustration, but over time the diagnosis enabled parents to find out more and indeed, many parents discovered the Goldenhar UK Charity. Uncertainty around the causation of Goldenhar was initially a problem, however, over time the lack of explanation was not pivotal; the supportive impact of the Goldenhar UK Group may have contributed to this. Doyle's (2014) [64] suggestion that interaction with others can mitigate against stigma and normalise common challenges could explain this. Feelings may have also dimmed with time.

All parents were extremely positive about the information and support offered by the Goldenhar UK Charity and Facebook group which provides a platform for sharing information, supplying reassurance, peer-support, and representation. Membership engendered feelings of empowerment in parents, including helping with decisions about surgery, dealing with societal reactions and supporting their children. This links with Bogart & Hemmesch's (2015) finding that repeated conference attendance correlated positively with improved emotional support and companionship [67].

Participants discussed experiencing discrimination and stigma at varying levels, including lower educational expectations, resonating with previous findings [40]. Parents are keen to raise awareness and move towards positive representation and normalisation of difference and this aligns with Changing Face's research which highlights that people with disfigurements are either hugely under-represented or misrepresented in the media [38]. Parents hoped the launch of the film and book *Wonder* would help tackle disfigurement inequality and raise awareness, although one parent criticized *Wonder* for using an actor without actual disfigurements. This view mirrors the perspectives of world-wide facial disfigurement and disability action communities' who criticised the film for ‘not representing the community it is supposed to be serving’. They believe for the media to effectively challenge the situation they need to broadcast people with real disfigurements [49].

The bombardment and obsession with “so-called” beauty-ideals and the focus on “perfection” was something that concerned parents as they feared their child may suffer discrimination or a loss of self-esteem. This supports media-brainwashing research which found the pressures young people experience to conform to the narrow ideals of so-called beauty is overwhelming, and undermines young people's self-confidence [88,89].

There appeared to be a consensus that acceptance of the condition occurred with time, Goldenhar had become *‘the new normal’* and after a while parents viewed their child just like any other child; challenges and obstacles had become normalized. Many parents reflected upon the fact that Goldenhar had changed them and *‘enriched’* their lives. Despite the initial shock and continuum of uncertainty, parents viewed their futures positively.

### 4.1 Strengths

By adopting a qualitative approach, the study provided a realistic account of the lived experiences and feelings of Goldenhar parents. The study included an equal balance of mothers' and fathers’ perspectives. Also, parents came from different locations across the UK, and therefore were supported by different paediatricians, medical professionals and multidisciplinary team members, providing a range of experiences to the sample.

We adopted an inductive-realist approach [76], which facilitated in highlighting the real-life issues Goldenhar parents face. Also, the semi-structured interview approach was extremely advantageous as it allowed for unplanned discourse. In addition, the interviews were conducted by a researcher with lived experience of parenting a child with Goldenhar. This familiarity undoubtedly aided the recruitment of participants and may have led to greater levels of participant disclosure, removing barriers between the researcher and the researched [90].

### 4.2 Limitations

In terms of limitations only married parents put themselves forward for interview and thus, we were unable to account for the views of single or co-habiting parents. Furthermore, all interviews were retrospective, where participants were asked to recount and make sense of their experiences. A disadvantage of this is experiences inevitably vary on a daily/monthly basis, therefore it is impossible to get a true picture. Also, parents were at different stages of their journeys, children varied in age and it may have been difficult for parents to remember accurately how they felt post-birth and in the early stages. Whilst the relatively small sample size could be seen as a limitation; it is important to remember this is a very rare condition and therefore recruitment of five sets of parents in the UK is representative of the numbers affected.

### 4.3 Conclusion

This is the first time the life perspectives of parents raising a child with Goldenhar Syndrome, have been explored via interviews. We have unearthed prominent issues that impact parental QoL including isolation and distress at the point of diagnosis. It is clear parents require information and support from initial diagnosis, and throughout the multidisciplinary health journey. We also have significant indicators of the ongoing QoL challenges faced by young people with Goldenhar Syndrome. These findings highlight the need for: more emotional support for parents in the initial stages; clear, supportive information for parents and practitioners to help mitigate against confusion, anxiety and isolation. Further, more research is required into Goldenhar causation, increased educational information sharing opportunities at Goldenhar UK Conferences, more public awareness and positive media representations of disfigurement promoting face-equality and inclusion.

### 4.4 Future implementations

A planned future-step, which is currently underway, is to use some of the study data to create downloadable Goldenhar UK information booklets for parents, families, and medical practitioners. The purpose of these information booklets is to inform and reassure new Goldenhar parents and ‘give voice’ to the parents and children affected by the condition. It is hoped the booklets will give hope, aid future understanding and break down societal stigma surrounding difference.

Lastly, these findings have led to a future programme of work which is underway, exploring the QoL issues Goldenhar presents for teenagers, young people and adults. The aim is to develop a conceptual framework of their QoL needs. This will be used to develop a bespoke patient reported outcome (PRO) to give voice to the challenges that children, young people and adults face during their medical journey.

## Financial statement

The study received no external funding.

## Data availability statement

Data in the present study are available upon request from the corresponding authors. The data are not publicly available because of privacy concerns and local ethics policies.

## Additional information

No additional information is available for this paper.

## CRediT authorship contribution statement

**Rebecca Hitchen:** Writing – review & editing, Writing – original draft, Methodology, Investigation, Formal analysis, Data curation, Conceptualization. **Maxine Woolhouse:** Writing – review & editing. **Patricia Holch:** Writing – review & editing, Supervision, Methodology.

## Declaration of competing interest

The authors declare that they have no known competing financial interests or personal relationships that could have appeared to influence the work reported in this paper.
