# Supplementary material for: Breaking the silence: A qualitative exploration of parental perspectives of children with Goldenhar Syndrome
Source: Heliyon. 2024 Jan 21;10(3):e24328. doi: 10.1016/j.heliyon.2024.e24328 (PMC10839885; doi:10.1016/j.heliyon.2024.e24328)
Supplement: Multimedia component 1 [file mmc1.docx]

**Supplementary information Table 1: Identified Themes, sub-themes and codes/minor themes**

| **THEME** | **SUB-THEMES** | **CODES/MINOR THEMES** |
| --- | --- | --- |
| 1. **Goldenhar UK – a lived experience network** | **1a) information and guidance – ‘our own encyclopedia’**  **1b) Emotional reassurance and ‘identification’**  **1c) Altruistic members –journey sharing and offering future directions.** | - A vital lived experience network – our own little google. - GH is a complex area –learning happens through world-wide sharing of information from parents, children and professionals - Goldenhar UK conferences and online fb answer specific question and queries. - Up-to-date practical information and advice given and professional support offered. - Information exchanges allow parents to compare services and medical advice across the UK - Offers parents an insight into the future. - Group support with surgical processes, operation and recovery. - New parents sceptical and scared about their first visit then met happy people – open to questions, sharing information – positive experience. - Seeing children smiling and happy at the GH weekend changed Jules’s outlook. - GH Conference benefits are invaluable for all the family. Support for parental anxiety, validation for children. - Children are excited about reuniting with friends at the conference. - Support from group leader – reduction in anxiety --acknowledgment of ups and downs. - Mutual understanding – on the same journey. Confidence-building and identification for GH children and parents. - Children can identify with others who look different like them or have had operations (FB online offered identification – a boy who looked like his son) - Identification with others lessens feelings of isolation and alienation. - Important friendships formed – friends understand more than parents/adults. - Children and parents feel as ease in the group – Goldenhar is normal. Confidence boost for children and parents - Peer mentoring - worry sharing -sharing experiences can be comforting. Bonds built through mutual understanding. - Support networks aid understanding and wellbeing – reduction in the feeling of mother’s guilt - Reassurance from others/prior knowledge – helps with shocks or things to watch out for – e.g unprofessional surgeon) - Changing Faces offer psychological telephone support. Scenario Support given. - Positive future outlooks. - Opportunities to share your knowledge   - Information/progress through the group. - Parents aspire to help new and younger parents cope with the initial shock, confusion and public interrogation. - (Desire to support/inform other GH parents how to get help at school) - Existing GH parents helping and supporting each other all at different stages) - Reading on fb online and helping others – the desire to give back - First-hand medical experiences and parental expertise informing other parents about things consultants don’t tell you. - Plans for the group’s future – 1) Fundraising ideas. 2) raising awareness – the group’s new information booklet drive -wanting to give back – group contributions and writing as therapy. 3) Continuing media awareness & hopes new film Wonder will help raise awareness in the media. 4) inviting older people with GH to the charity to share experience. Valuable life experiences older GH sharing with younger & 5) more peer mentoring. |
| 1. **Gendered differences in coping** | **2a) Gendered emotional responses**  **2a) Did Gendering of roles come into play?** | **Fathers**   - Chloe’s husband found the 1^st^ week more harrowing than she did – he cried every night and she soldiered-on. - Bruce felt scared and robbed of his experience of being a new father - Intense fear of problems meant that Bruce missed his daughter’s birth - Bullying was Jules’s biggest initial fear - Jules said long periods at the hospital and waiting to get out was emotionally tough - Michael was not worried about the difference - more worried about the low birth weight and other people’s reactions. - (Michael supported wife with guilt, no known cause-helped his wife. - Luke –focused on his baby’s and wife’s well- being – not differences. Proud dad – always focused on the child not the condition. - Maximus emotional response was it’s fine differences happen. He said he adopted a pragmatic approach and his wife was emotional.   **Mothers**   - Chloe soldiered-on – quickly accepting the difference. - Sonia felt angry, frustrated and defensive about unwanted comments/interrogation. - Judy said the unknown combined with grief sent her a bit crazy initially – she wanted answers. She Initially panicked about what GH meant and thought catastrophically about the impact it would have on their families future. - Initial inability to identify/ relate to other mum’s and frustration over other mum’s complaints about ‘minor’ health issues - Guilt mother-blaming. Jasmine felt guilt and overwhelmed. Other people’s reactions made her feel guilty. - Lois blamed, questioned herself & analysed everything –other mothers did this. - Judy and other mums felt conflicted by the fact that they put off motherhood for a career, travel etc) - Lois’s- lack of emotional support – struggled to cope. - Sonia struggled to cope with the confusion and the new role of mother/carer. Lack of support and the loss of freedom – stress. - Emotional distress put pressure on Judy’s marriage and finances were a concern. - Chloe puts pressure on herself to hide and suppress anxiety.   **Fathers**   - Luke’s had a masculine approach– not affected emotionally. Focused on his wife’s wellbeing & the bigger picture. He adopted a pragmatic, positive, forward-thinking, empathetic approach – solving problems, communicating and supporting his wife and daughter. Luke gendered emotion saying mums worry more. - Maximus -said he and his wife had different reactions his wife was emotionally driven and he adopted a clinical approach – getting on. Emotional feeling supressed - focus on fact finding and pragmatism) One day at a time. - Jules found the GH support group and soon had a positive outlook. Jules’s wife did research on Goldenhar UK. - Bruce and his wife – worked together as carers initially. - Michael reassured his wife and adopted a philosophical outlook.   **Mothers**   - Judy considered gendered differences to coping – differences were complimentary) Gendered differences – father more pragmatic, mother more emotional but able to deal with the practical aspects feeding etc. Gendered differences – Judy said her husband struggled with the reality of surgery. - Jasmine was responsible for the appointments but was supported. - Chloe took the practical approach. She researched the condition and diagnosed it. Chloe could confidently explain Goldenhar to her husband and others - Chloe experienced subtle pressure from a family member to give up her job but Chloe’s husband recognized the importance of her job. - A work-life balance is hard to judge and guilt felt by mothers. Judy felt guilt about her job. - Sonia & Bruce split the care roles and Sonia took on the protective role. |
| **3) A rollercoaster of emotion** | **3a) Feelings at birth ‘ a whirlwind of emotion’**  **3b) Goldenhar –post diagnosis**  **3c) Dealing with other people’s reactions** | - Total shock - nothing detected in the scans! Confusion –no warning signs in pregnancy or pre-birth. No indicators in pregnancy-shock - Panic stations/ a hive of activity)   - An overwhelming catalogue of shocks and differences identified. Shock of taking blood, scans, tests – the unknown - Overwhelming confusion – what is it? - Initial feelings – anger, disbelief, strong feeling of guilt and blame –wanting answers – why? what! Why? we didn’t do anything!? - Initial reactions were grief, physical pain, shock & disbelief, sorrow, questioning. Anger at the world -– things felt unfair - Feelings of failure – disappointing others   - Self-blame/ self-doubt - Isolation and alienation –wanting to hide the child and protect themselves) - Initial feelings of the long haul. Hindsight suspected something was wrong at birth. - Frustration over assumed medical knowledge – panic about what will be next. Uncertainty – living for the next 24 hours - Struggling to feed was stressful. - Diagnosis timescales varied from diagnosis at birth to diagnosis after 18 months. - Denial – this is not happening to me- Scared and in disbelief. - Overwhelming confusion – nobody knew where to start. Lack of information – frustration - Jukes was scared for his son and scared of judgement from others. - Distance from others. The desire to isolate oneself. - Self-blame/guilt, disappointment - Shock and emptiness. Lack of support struggled to cope - Judy Initial panic about what GH meant and catastrophic thinking over the impact it would have on their future lives) - Jules Fear of bullying/not wanting a difference - The unknown combined with grief send you a bit crazy initially - At first difference meant isolation then tube-feeding in Nero became the new normal. - Diagnosis helped but still a life changing condition. Luke said it was a difficult situation but grateful for his child conceived through IVF) - Being a parent and a nurse is challenging – an emotional rollercoaster. - Difference brings challenge - Life is up and down. - The strive for normality and normal socialization is strong. - Being stared at was hard. - unkind reactions from neighbours. - Other people’s ignorance/ lack of understanding angered Judy. - Dealing with other people’s questions was difficult. Feeling forced to provide information to others - Talking on demand was emotionally draining - Invasion of privacy - constant interrogation over difference. - Jasmine said others reactions led to feelings of anxiety and self-blame - Lois said subtle stigma felt led to feeling of anxiety and the desire to isolate. - Jules resilience to looks from other– laughs it off. |
| **4) Uncertainty** | **4a) Lack of clarity over causation**  4b) ‘An uncertain journey.’ | - Lack of clarity about the difference in conditions – definitions hemi-facial microta and GH) - Causation is totally unclear - No common ground – no links - Lack of clarity – wanting answers - Searching for an explanation, Self-questioning and looking for why is a dominant feature initially and causes guilt - speculation happens - No known cause-helped wife with guilt lack of clarity – no reason no blame - Confusion- explanation would help with acceptance) - Clarity would aid understanding but not change the outcome for their child. - Lack of clarity – uncertainty over future family planning for parents. - Paranoia and uncertainty felt in second pregnancy No additional tests given for daughter – no differences in pregnancy. - Father was so scared and uncertain - that he missed his daughter’s birth. - Clarity would reassurance GH kids for their futures. - Currently parents have no other choice than to live with the uncertainty of causation. - 2x dads – we’ll never find out why – it doesn’t matter - Others hope the uncertainty may eventually be uncovered) hopefully a future discovery**.** - You never know what’s next - Uncertainty over the extremity of GH and the progression of the condition -the doctors are uncertain - Uncertainty over test results –Constant uncertainty and anxiety when waiting for test results. Hitting ‘so called’ milestones were highly reassuring – positive results were a relief. - Medical time-scales are unknown and mental preparation. - Uncertainty about how their children will cope. - Will their anxiety of the unknown be projected onto their children? - Uncertainty over the reactions of siblings - Future fears – uncertainty about the prospect of bullying and integration. - Uncertainty over parental intervention & giving confidence to their child. uncertainty about defence techniques) - Explaining to your child why they have GH – uncertain how. - Anxiety/uncertainty about adolescence son’s future relationships/ family life chances) Wanting reassurance from older Goldenhar people - Uncertainty about child’s wellbeing & teenage worries – changes in moods. Worry about daughter’s anxiety/ self-esteem) - Surgical decisions are complex, time consuming, anxiety provoking and overwhelming – what is the right decision? - It is difficult to make an informed decision -there is insufficient comparable data and no cohorts to make comparisons with - Surgical decisions for aesthetic reasons are harder- worry about decision making & daughter’s wellbeing, future/teenage body image – daughter starting to obsess). - It is hard passing the decision responsibility to a child – anxiety and pressure - Parents made to feel guilty about decision making. Pressures from surgeon and others about decision making. - Parents are uncertain how to put personal biases to one side. - Uncertainty and surgery worries put pressure on the family. The child may not want it –what is right? |
| **5) Societal reactions** | **5a) Public ignorance and stigma**  **5b) Raising awareness**  **‘We are all people regardless of difference’.**  **5c) Challenging society’s idealized views of beauty.** | - People are uncomfortable and unsure how to respond and look away. - Rude, ignorant reactions from the public. - Ignorance from other children – led to a few hurtful comments initially. - Jules’s boy often mistaken for a girl because he has long hair - Pubic abuse and prejudice happens – people talking down - Other people are shocked when they hear about the condition. - Assumptions made by the public. The public have drastic pictures in their head and (pre-conceived ideas) - Subtle stigma felt by Lois - socialising became more difficult) - Lois felt the judged/blamed - people speculating over causation. - Other people’s reactions led to mother’s feelings of self-blame. Emotionally draining, feeling defensive - Parental anger felt in reaction to other’s looks and judgements. - Parents looking for strategies to stop the staring and questioning. - Ignorance led to the making of information cards helped fend off unhelpful comments - Other people’s reactions led to mother’s feelings of self-blame. - Friends don’t understand - Socialising reduced due to caring needs - lack of understanding from friends. - Other parents are unaware of the challenges that GH parents face - More blame and stigma felt by mothers - Fathers – felt less stigma other people look – stigma not felt. - Other people’s lack of awareness because of lack of experience. - People stare because they are curious. Judy would like to inform more people and perceives stares as curiosity rather than anything malicious. - Assumptions made/ People are unaware and need educating to avoid assumptions & discrimination ( talking down) - More awareness needed to understand and respond to difference – more information needed - relaxed q& a session – it is normal to notice difference and talk about it. - Focus on normal socialization and integration. Look for similarities not differences Many non-GH siblings have made good friendships with children with facial differences. - One child used a school awareness strategy –letter to parents) - Need more focus on E&D - (Parental outlook –don’t judge – treat others well) - There is lack of media representation. More media awareness/publicity needed to educate people. - Recent initiatives – CBC Normalisation in the media needed – difference is accepted now. - changing faces – fb posts - The new blockbuster Wonder should help to increase awareness. - (More actors with disfigurement needed) If the main Wonder actor had disfigurement in real life then it would be more meaningful. - Good ideas -fundraising and our Goldenhar booklet plan. - Societal ideology and the mass media create pressure and conflict with unrealistic body types. - Unrealistic images and suggesting a need for change has a psychological impact. - Chloe sees the media’s focus on idealized beauty as a threat to her daughter’s well-being. - Expectation of a ‘perfect baby’ Societal obsession with the perfect baby - Lack of regard/empathy for people who have a child who is ‘not perfect’ - The notion of perfection can have negative connotations - Societies focus on perfection is irritating! - Philosophical father, Michael, says Societal ideals of beauty – focus on perfect. It is unfair and unequal. Concerns over daughter’s self-esteem. - (More actors with disfigurement needed) - Facial inequality ideals oppress people |
| **6) Coping with challenge** | **6a) Parental coping strategies/ dealing with the emotional burden.**  6b) Supporting our children | - Don’t tell the full story - just tell other parents about one aspect e.g. feeding - Use one liners to cut people short– other people don’t need to know everything. - GH cards offered a solution to interrogation. - Go-with-the-flow more - Live for now – one day at a time. Don’t look too far into the future. - Do mental preparation before operations – prepare for anxiety, nil by mouth etc - Have patience and look for new strategies - Intrusion and public ignorance is still out there - ignore it. - Smile and carry on - show strength –looking is no big deal) - Open approach to questions breaks down barriers. - Keep things in perspective it is all on a scale - Have an optimistic outlook about surgery – don’t meddle yourself with worry. One step at a time - Be open to questions and direct with people **-** Anxiety about what others think will reduce over time. - Allowing children to make their own decision is hard but go with it. - Stress is a continuum – don’t let GH stresses dominate - Focus on the positives, be grateful. - Friendship circles may change now more time spent with friends who have children with differences. - Spend time with accepting friends/family – no prejudice – spiritual support/acceptance - Look back and know you did your best - Strong parental role-models needed and mechanisms to deal with potential bullying. - Family conversations support and prepare children for the future**.** - Use of distraction techniques –they are looking at your t-shirt. Change techniques over time) - Giving children phrases they can use – equipping them to answer and educate others. - Bruce’s son taking responsibility – fighting against discrimination. Inform others before they get the chance to criticise/ assume or take control. - Keep your defences up – not letting others take over. - Luke’s daughter wrote a letter to school parents -School coping strategy) - Parents taught strategies to fight against bullies) - Jules –is teaching strength of character – don’t let things bother you. - Judy adopted an honest matter of fact approach. - Forward thinking, positive approach to life. Instils confidence in children. - Coping = breaking things down – one step at a time for children -decision making for children can cause anxiety - Remember that making sense of the world is a complex and timely journey for children. |
| **7) Retrospective views** | **7a) Acceptance ‘Goldenhar the new normal.’**  **7b) A change in perspective- ‘a positive outlook’.** | - Acceptance happens with time – all at different stages of the journey. - Diagnosis helped slightly but so did time. Diagnosis helped with feelings of blame - Blame diminishes with time and greater understanding and acceptance occurs. Why becomes less important. - Coming to terms with life changes, loss of freedom and time and many appointments. - Delay in joining the group -need to accept the situation personally first. - Acceptance & normalisation with time – child treated the same as sibling - Goldenhar has become the new-normal – Maximus – No challenge– just needs support with speech – support assessed Goldenhar is normal – difference is fine. - Goldenhar has become so normal that often Judy forgets he has it. - Older sibling helped with life perspective - ‘you have to get on.’ - Judy -optimism continued after the initial shock, normalisation occurred with equal worrying about both children. - There are times when we can live a normal family life without any major decisions (Jas) - Luke’s daughter’s behaviour was normal, normal relationships formed (strong friendships) - In retrospect it was clearer to see it was nothing anyone had done. - Strong parental bond/love grows stronger – (differences don’t matter)) - Jules’s son has proven him wrong – his initial thoughts about his son being bullied have changed – now feeling proud) (Proud Father – child exceeding all initial expectations) - Bruce is proud of his son’s achievements and ability to overcome obstacles. - Coming to the group has changed parents outlook including RH’s - Now future optimism/ open-mindedness. - Roles shifting from mum to carer freedom increasing over time. - Resilience and strength - stronger family as a result, changed life perspective – scared changed to proud dad - Goals, expectations and perspectives have shifted – time for each other and positivity are important. - Ideals of family life have changed – Mary Poppins ideals have gone. - Extra life considerations have made me a better person (Chloe). - The difference/challenge has changed Judy and enriched her life as she has many amazing people. Life is unpredictable - Having a brother with a difference has made her more understanding and empathetic towards others – a positive outcome) - Positive outlook - wouldn’t change a thing. |
